# Interaction analysis on transmissibility of main pathogens of hand, foot, and mouth disease: A modeling study (a STROBE-compliant article)

**DOI:** 10.1097/MD.0000000000019286

**Published:** 2020-03-13

**Authors:** Kaiwei Luo, Jia Rui, Shixiong Hu, Qingqing Hu, Dong Yang, Shan Xiao, Zeyu Zhao, Yao Wang, Xingchun Liu, Lili Pan, Ran An, Dongbei Guo, Yanhua Su, Benhua Zhao, Lidong Gao, Tianmu Chen

**Affiliations:** a Hunan Provincial Center for Disease Prevention and Control, Changsha, Hunan Province; b State Key Laboratory of Molecular Vaccinology and Molecular Diagnostics, School of Public Health, Xiamen University, Xiamen, Fujian Province, People's Republic of China; c Division of Public Health, School of Medicine, University of Utah, Salt Lake City, UT; d Changsha Center for Disease Prevention and Control, Changsha, Hunan Province, People's Republic of China.

**Keywords:** hand, foot, and mouth disease, interaction, mathematical model, transmissibility

## Abstract

Hand, foot, and mouth disease (HFMD) has spread widely and led to high disease burden in many countries. In this study, we aimed to analyze the interaction of the main pathogens of HFMD using a mathematical model.

A dataset on reported HFMD cases was collected from April, 2009 to December, 2017 in Changsha City. A long-term etiological surveillance was conducted focusing on the pathogens of the disease including enterovirus A71 (EV71), coxsachievirus A16 (CA16), and other enteroviruses. A susceptible-infectious-recovered model was adopted to calculate the reproduction number during the ascending period of reported cases (defined as *R*_*asc*_) and the descending period (defined as *R*_*des*_).

About 214,178 HFMD cases (including clinically diagnosed cases and confirmed cases) were reported in Changsha City, among which 31 were death cases with a fatality of 0.01%. The number of reported HFMD cases increased yearly with a Linear model of “*f*(*t*) = 18542.68 + 1628.91*t*” where *f*(*t*) and *t* referred to number of reported cases and sequence of year, respectively. The fatality of the disease decreased yearly with a linear model of “*f*(*t*) = – 0.012 + 0.083/*t*”. About 5319 stool or anal swab specimens were collected from the reported cases. Among them, 1201 were tested EV71 positive, 836 were CA16 positive, and 1680 were other enteroviruses positive. *R*_*asc*_ and *R*_*des*_ of HFMD was 1.34 (95% confidence interval [CI]: 1.28–1.40) and 0.73 (95% CI: 0.69–0.76), respectively. EV71 and CA16 interacted with each other, and the interaction between EV71 and other enteroviruses and the interaction between CA16 and other enteroviruses were both directional. However, during the reported cases decreasing period, interactions only occurred between EV71 and other enteroviruses and between CA16 and other enteroviruses. These interactions all decreased *R*_*asc*_ but increased *R*_*des*_ of affected pathogens.

The interactions of the pathogens exist in Changsha City. The effective reproduction number of the affected pathogen is adjusted and verges to 1 by the interaction.

## 1 Introduction

Hand, foot, and mouth disease (HFMD) is a common infectious disease and has spread widely in China and in many other countries, such as Malaysia, Thailand, Vietnam, Cuba, and India.^[1–7]^ Traditionally, epidemiological studies are focusing on describing incidence and the demographic characteristics (age, sex, and so on) of the disease. However, those epidemiological characteristics are based on the transmission force or the transmissibility of the disease. Reproduction number, which is defined as the expected number of secondary infections that result from introducing a single infected individual into an otherwise susceptible population,^[8,9]^ is commonly employed to quantify the transmissibility.

Many mathematical models, including susceptible–infectious–recovered (SIR), time series SIR, susceptible–exposed–infectious–recovered (SEIR), susceptible–exposed–infectious–quarantined–recovered (SEIQR), and susceptible–exposed–infectious–asymptomatic–recovered–environment (SEIARW), have been employed to calculate the reproduction number of HFMD.^[10–15]^ The reproduction numbers of enterovirus A71 (EV71) and coxsackievirus A16 (CA16) had also been estimated.^[16]^ To explore the transmission mechanism, our previous research analyzed the epidemiological characteristics with a new method by calculating a similarity index,^[17]^ calculated the production number of the HFMD in county level,^[18]^ estimated the relative transmissibility between male and female,^[19]^ and simulated the seasonality of the transmissibility of the disease.^[20]^ However, the interaction on the transmissibility of the main pathogens of HFMD remains unclear.

In this study, we assumed that the transmissibility of HFMD's main pathogens is different, and interaction on the transmissibility of the main pathogens exists. Therefore, we collected the data on reported HFMD cases in Changsha City. A long-term etiological surveillance was conducted in the city to calculate the incidence of EV71, CA16, and other enteroviruses. A SIR model was adopted to calculate the transmissibility and its interaction of the 3 pathogens.

## 2 Methods

### 2.1 Ethics statement

This effort of outbreak investigation and control was part of routine responsibility of Hunan Provincial Center for Disease Control and Prevention (CDC). This study was approved by the Ethics Committee of Hunan Provincial CDC. All data analyzed were deidentified.

### 2.2 Study sites and data collection

In this study, Changsha City in Hunan Province was selected as study site. Changsha City, the capital city of Hunan Province and is located in central south China, has a population of more than 7 million and includes 6 districts, 2 county-level cities, and 1 county.

Two main datasets were used in this study. The first one (Dataset A) included the weekly number of reported cases and death cases from April 2009 to December 2017. The cases were classified as clinically diagnosed cases and confirmed cases. The second one (Dataset B) was based on an etiological surveillance which was aimed to understand the transmission of the pathogens of HFMD. The etiological surveillance was conducted from 2009 in the city. Each month, 5 stool or anus swab samples were collected from each clinically diagnosed case in each district or county. The samples were then sent to the laboratory of municipal CDC to identify the pathogens (EV71, CA16, and other enteroviruses denoted as “Other”) of the disease by polymerase chain reaction. The proportion of the 3 pathogens was therefore calculated to estimate the numbers of HFMD cases infected with EV71, CA16, or other enteroviruses. Based on the etiological surveillance data, the number cases of EV71, CA16, and Other were adjusted for estimating the transmissibility of the 3 pathogens of HFMD. The equation for calculating the adjusted cases of EV71, CA16, and Other was shown as follows:
 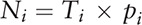


In the equation, *i* = 1, 2, 3 which refer to EV71, CA16, and Other, respectively. And *N*, *T*, and *p* refer to the number of adjusted HFMD cases resulted from the specific pathogen (EV71, CA16, or Other), the total number of reported HFMD cases with all the pathogens, and the proportion of each pathogen based on the Dataset B, respectively.

In addition, population of the city, birth rate, and death rate data were collected from statistical yearbooks announced by the statistical bureau of the city.

### 2.3 Simulation model and transmissibility estimation

According to our previous study,^[18]^ a susceptible-infectious-recovered (SIR) model was adopted considering the natural birth rate and death rate of the population. The equations of the model were shown as follows:
 
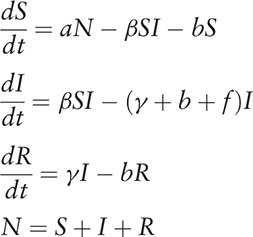


In the equations, *S*, *I*, *R*, and *N* refer to susceptible individuals, infectious individuals, recovered individuals, and number of the whole population, respectively. Parameters *a*, *b*, *f*, *β*, and *γ* refer to natural birth rate of the population, death rate of the population, fatality of HFMD, transmission relative rate, and recovered relative rate, respectively.

The reproduction number was utilized to quantify the transmissibility of HFMD. We assumed that the reproduction number of the disease during the ascending period of reported cases (defined as *R*_*asc*_) was different to the one during the descending period (defined as *R*_*des*_). They were calculated by the following equations:
 
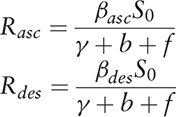


In the equations, *β*_*asc*_ and *β*_*des*_ referred to transmission relative rate during the ascending and descending period, respectively.

### 2.4 Parameter estimation

Parameters *a*, *b*, and *f* were calculated from the collected data. According to the published study,^[10,14]^ the infectious period of HFMD was about 2 weeks, therefore *γ* = 0.5.

From the Dataset A, the weekly number of HFMD cases was calculated to analyze the epidemic of the disease, and an epidemic peak was divided into ascending and descending parts which were employed to fit the SIR model to calculate *β*_*asc*_ and *β*_*des*_ in each epidemic peak. The values of *R*_*asc*_ and *R*_*des*_ were calculated consequently. The method of calculating *R*_*asc*_ and *R*_*des*_ has been published in our previous research.^[18]^

### 2.5 Interaction of the three pathogens

Based on the etiological surveillance data, the adjusted numbers of cases of EV71, CA16, and Other were calculated. Therefore, the values of *R*_*asc*_ and *R*_*des*_ were estimated in 7 scenarios (EV71, CA16, Other, “EV71+CA16”, “EV71+Other”, “CA16+Other”, and “EV71+CA16+Other”).

If the transmissibility of one pathogen was higher than that under the scenarios that another pathogen or more pathogens transmit at the same time, we could conclude that the other pathogens decrease the transmissibility of the pathogen. For example, if *R*_*asc*_ of EV71 was higher than *R*_*asc*_ under the scenarios that “EV71+CA16,” “EV71+Other,” and “EV71+CA16+Other,” we could conclude that CA16 and Other decrease the transmissibility of EV71.

### 2.6 Statistical analysis

Berkeley Madonna 8.3.18 (developed by Robert Macey and George Oster of the University of California at Berkeley. Copyright 1993–2001 Robert I. Macey & George F. Oster) was employed to run the SIR model and least root mean square (RMS) was adopted to assess the best candidate model provided by the software. While the curve fit is in progress, Berkeley Madonna displays the RMS deviation between the data and best run so far. The deviation is RMS of the differences between individual data points in the dataset and the corresponding points in the model run. Berkeley Madonna only shows the results of the best run. However, RMS could not tell how much percentage of the reported data the model can explain and whether the difference between the reported and simulated dataset is statistically significant. If the dataset run by the model has no significance with the reported dataset, we could conclude that the relationship between simulated data and reported data is simple linear regression. Based on the published literature,^[21]^ and our previous researches,^[8,20]^ determination coefficient (*R*^2^) could be employed to test the goodness of fit of the curve fitting.

SPSS 13.0 (IBM Corp, Armonk, NY) was employed to analyze the difference and the interaction of *R*_*asc*_ and *R*_*des*_ among the 3 main pathogens of the disease.

Eleven models (Linear, Logarithmic, Inverse, Quadratic, Cubic, Power, Compound, S, Logistic, Growth, Exponential, and Logistic) in SPSS 13.0 were employed to fit the yearly trends of reported HFMD cases and fatality. The equations of the 11 models were shown as follows:
 
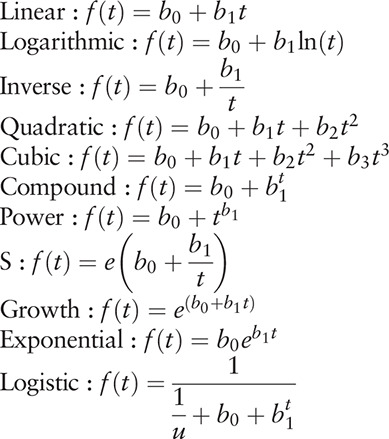


In the equations, *f*(*t*) refers to the dependent variables which are reported yearly incidence of HFMD and yearly fatality of the disease, and *t* refers to the time (year).; *b*_0_, *b*_1_, *b*_2_, *b*_3_, and *u* refer to the coefficients of the models which were estimated by curve fitting with the data. *R*^2^ was employed to evaluate the curve fitting.

## 3 Results

### 3.1 Epidemiological features of the disease

**Figure 1:**
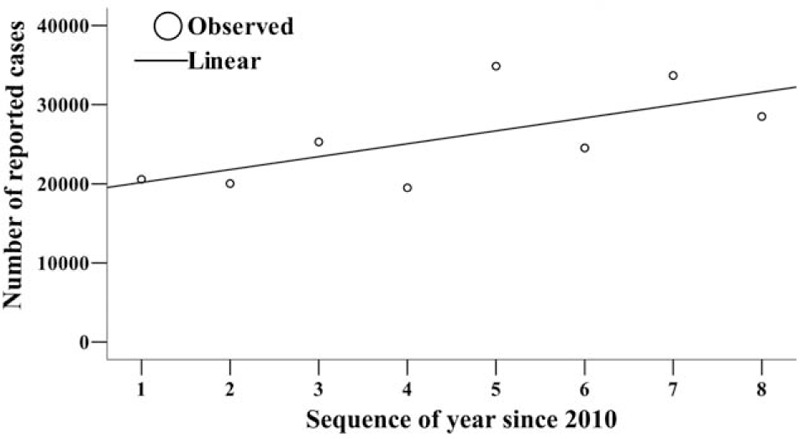
Yearly number of reported HFMD cases and its trend in Changsha City.

**Figure 2:**
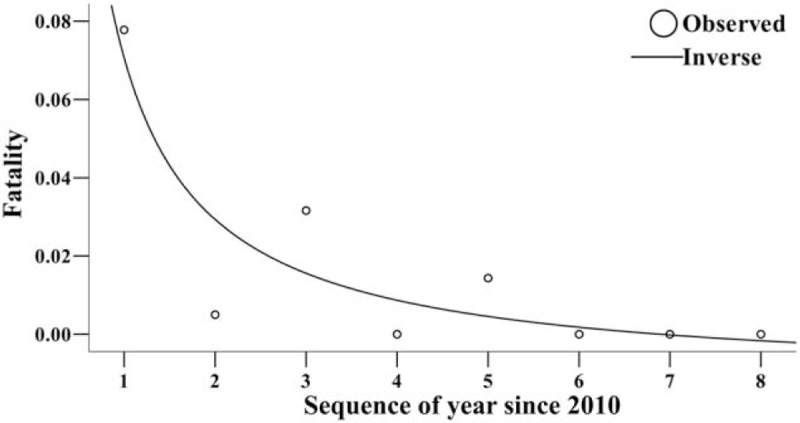
Fatality of HFMD and its trend in Changsha City.

From April 2009 to December 2017, 214,178 HFMD cases (including clinically diagnosed cases and confirmed cases) were reported in Changsha City, among which 31 were death cases with a fatality of 0.01%. Since the data from January and March in 2009 were not included, we used the data from 2010 to 2017 in Changsha City to fit the 11 models. The results of the curve fitting showed that the number of reported HFMD cases increased yearly with a Linear model of “*f*(*t*) = 18542.68 + 1628.91*t*” where *f*(*t*) and *t* referred to number of reported cases and sequence of year, although no statistical significance was observed (*R*^2^ = 0.439, *F* = 4.687, *P* = .074) (Fig. 1). However, the fatality of the disease decreased yearly. The best fitted model was the Inverse model, and its equation was “*f*(*t*) = – 0.012 + 0.083/*t*” where *f*(*t*) and *t* refer to the fatality and sequence of year, respectively. A statistical significance was observed (*R*^2^ = 0.793, *F* = 22.974, *P* = .003) (Fig. 2).

**Figure 3:**
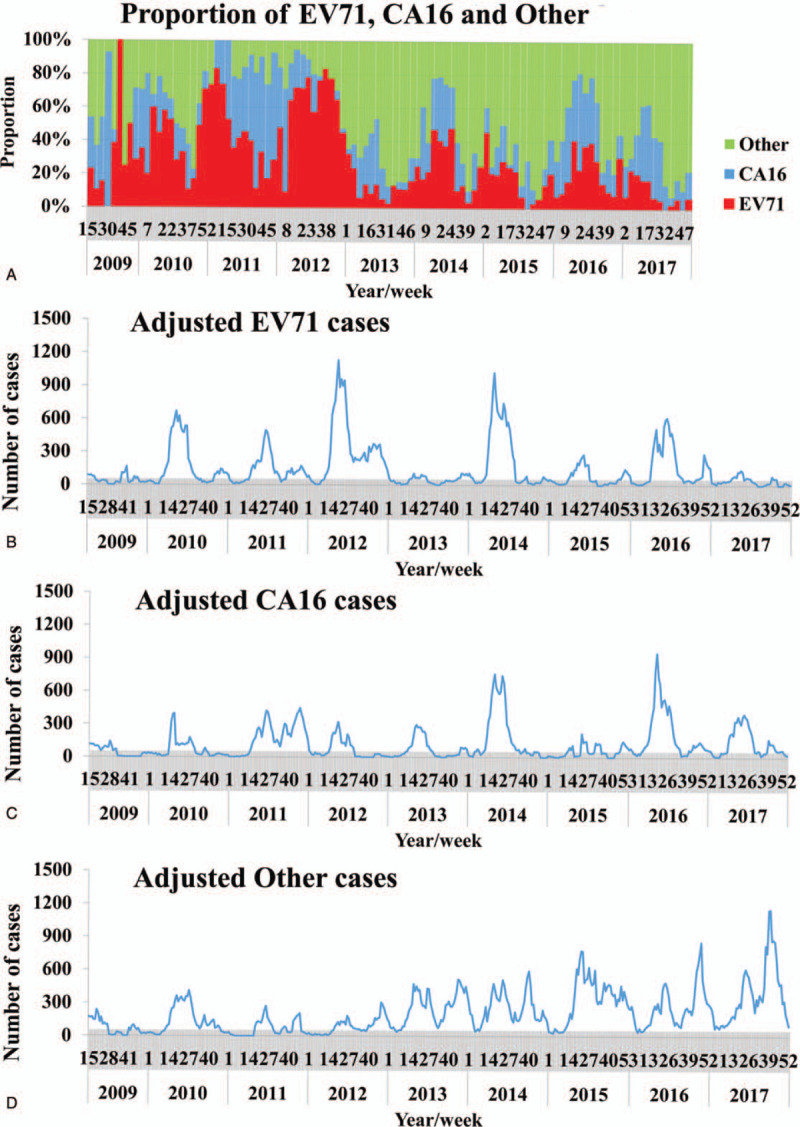
The temporal distribution of the proportion and the adjusted cases of the 3 pathogens of HFMD in Changsha City, 2009 to 2017. A, Proportion of EV71, CA16, and Other; B, adjusted EV71 cases; C, adjusted CA16 cases; D, adjusted Other cases.

From April 2009 to December 2017, about 5319 stool or anal swab specimens were collected from the reported cases, among which, 1201 samples were tested EV71 positive, 836 were CA16 positive, and 1680 were other enteroviruses positive. The temporal distribution of the proportion of the 3 pathogens was shown in Figure 3A. Based on the proportion, the adjusted numbers of cases for the 3 pathogens were calculated and were shown in Figure 3B, C, and D.

### 3.2 Transmissibility of the three pathogens

**Figure 4:**
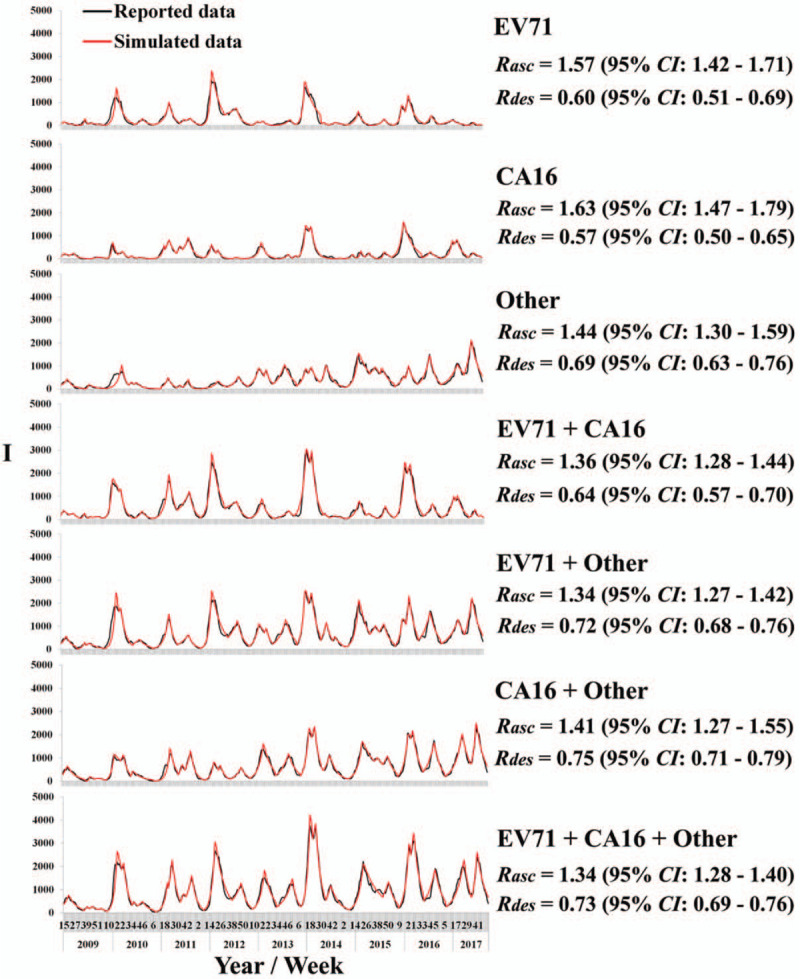
Results of model fitting and the transmissibility of HFMD's 3 pathogens in Changsha City.

The results of model fitting in Figure 4 showed that 26 epidemic peaks of HFMD cases were reported from April 2009 to December 2017 in Changsha City, with *R*_*asc*_ and *R*_*des*_ of 1.34 (95% CI: 1.28–1.40) and 0.73 (95% CI: 0.69–0.76), respectively. The numbers of epidemic peaks of EV71, CA16, and other enterovirus were 24, 29, and 24, respectively. The *R*_*asc*_ of EV71, CA16, and other enterovirus was 1.57 (95% CI: 1.42–1.71), 1.63 (95% CI: 1.47–1.79), and 1.44 (95% CI: 1.30–1.59), with no statistical significance of *R*_*asc*_ among the 3 pathogens (*F* = 1.527, *P* = .224). The *R*_*des*_ of EV71, CA16, and other enterovirus was 0.60 (95% CI: 0.51–0.69), 0.57 (95% CI: 0.50–0.65), and 0.69 (95% CI: 0.63–0.76), with no statistical significance of *R*_*des*_ among the 3 pathogens (*F* = 2.569, *P* = .083).

**Figure 5:**
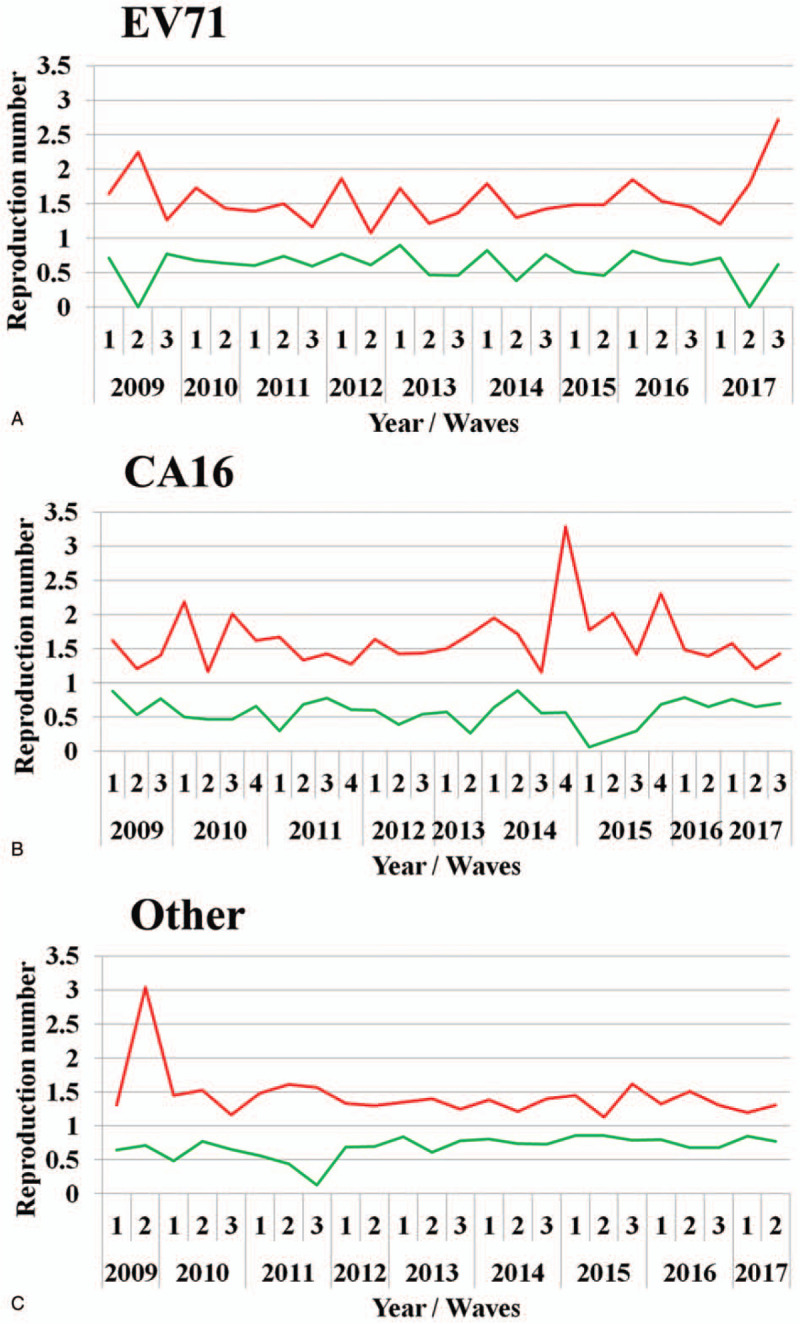
The trends of the transmissibility of HFMD. A, EV71; B, CA16; C, Other.

The transmissibility of the 3 pathogens kept in a stable level during the study period except some fluctuation of the transmissibility of CA16 from 2010 to 2011 in Changsha City (Fig. 5). The *R*_*asc*_ of EV71 increased in 2017. The *R*_*asc*_ of CA16 increased obviously from late 2014 to 2015. The *R*_*asc*_ of the other enteroviruses increased obviously in 2009 (Fig. 5).

### 3.3 Interaction of the three pathogens

To analyze the interaction of the 3 pathogens, we compared the transmissibility (*R*_*asc*_ and *R*_*des*_) between each other for the 3 pathogens (Table 1). *R*_*asc*_ values between EV17 (mean = 1.57) and EV17 + CA16 (mean = 1.36), EV71+Other (mean = 1.34), and EV71+CA16+Other (mean = 1.34) were all statistically significant. These results revealed that CA16 and Other could decrease the *R*_*asc*_ of EV71 when these pathogens transmit together. *R*_*asc*_ values between CA16 (mean = 1.63) and CA16+EV17 (mean = 1.36), CA16+Other (mean = 1.41), and CA16+EV71+Other (mean = 1.34) were also statistically significant, which revealed that EV71 and Other could decrease the *R*_*asc*_ of CA16 when these pathogens transmit together. However, no statistical significance of *R*_*asc*_ was observed between Other (mean = 1.44) and Other+EV17 (mean = 1.34), Other+CA16 (mean = 1.41), and Other+CA16+EV71 (mean = 1.34).

**Table 1:**
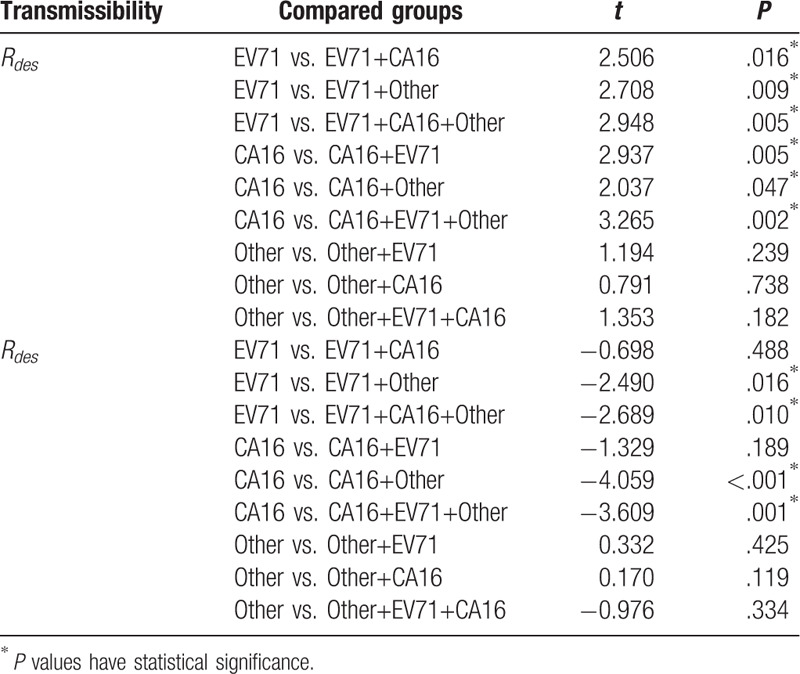
Interaction of transmissibility of HFMD's 3 pathogens in Changsha City.

*R*_*des*_ values between EV17 (mean = 0.60) and EV71+Other (mean = 0.72), and EV71+CA16+Other (mean = 0.73) were statistically significant, except between EV17 (mean = 0.60) and EV17+CA16 (mean = 0.64). These results revealed that Other could increase the *R*_*des*_ of EV71 but CA 16 could not when these pathogens transmit together. *R*_*des*_ values between CA16 (mean = 0.57) and CA16+Other (mean = 0.75), and CA16+EV71+Other (mean = 0.73) were statistically significant except between CA16 (mean = 0.57) and CA16+EV17 (mean = 0.64). These results revealed that Other could increase the *R*_*des*_ of CA 16 but EV71 could not when these pathogens transmit together. No statistical significance of *R*_*des*_ was observed between Other (mean = 0.69) and Other+EV17 (mean = 0.72), Other+CA16 (mean = 0.75), and Other+CA16+EV71 (mean = 0.73) (Table 1). These differences revealed that the interactions of the 3 pathogens were complex in Changsha City.

**Figure 6:**
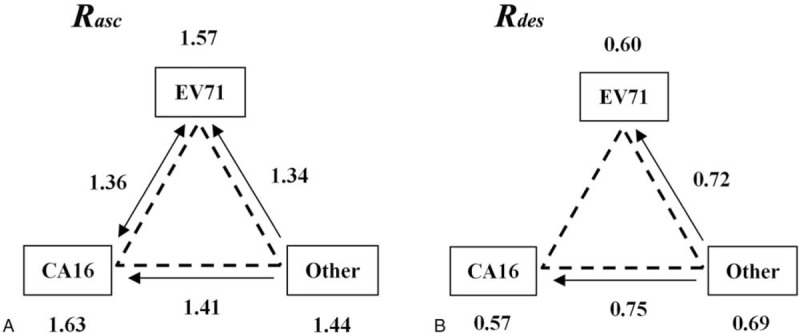
The interaction modes of the transmissibility of HFMD in Changsha City. A, *R*_*asc*_; B, *R*_*des*_. Arrow indicates the direction of interaction from one pathogen to another one; no arrow refers to no interaction.

During the increasing period of the number of reported cases, EV71 and CA16 interacted with each other, but the interactions between EV71 and other enteroviruses and between CA16 and other enteroviruses were both directional. These interactions all decreased the transmissibility (*R*_*asc*_) of affected pathogens (Fig. 6A). However, during decreasing period, interactions were only existed between EV71 and other enteroviruses and between CA16 and other enteroviruses. The transmissibility (*R*_*des*_) of EV71 and CA16 both increased when other enteroviruses spread in the same period (Fig. 6B).

## 4 Discussion

Although there are over 20 types of pathogens of HFMD,^[12]^ the main pathogens of the disease are EV71 and CA16.^[22–24]^ These pathogens are usually prevalent in the same area at the same time. Therefore, it is important to understand that whether these pathogens interact with each other. Although there was a research focusing on calculating the transmissibility of EV71 and CA16,^[16]^ however, to our knowledge, our research is the first study to estimate the interaction by using mathematical models. The results of the test of goodness of fit showed that our models fit the reported data well, which meant that our modeling procedure had good validity, and the models could be used to calculate the transmissibility of each pathogen.

In our study, we found that the number of reported cases increased but fatality decreased yearly in the city. The decreased fatality might be due to the enhanced surveillance system and improved diagnosis and treatment ability of health departments, clinics, and hospitals. The increased incidence revealed that the pathogens of HFMD were still spreading in the city.

Considering that the effectiveness of interventions existed during the reported cases descending period, we recommend that the transmissibility of HFMD could be estimated by using *R*_*asc*_. Calculated by the SIR model, we found that the transmissibility (*R*_*asc*_) of EV71, CA16, and the other enteroviruses in the city was 1.57, 1.63, and 1.44, respectively. These results indicated that the transmissibility of HFMD was lower than influenza. The effective reproduction number of influenza was 1.4 to 2.0,^[25]^ and was 1.81 in Changsha City.^[26]^ Therefore, the results of our study are reliable. According to the stable trends of the transmissibility of the 3 pathogens of HFMD, the transmission force would keep in a stable level, thus we could make an evidence-based prediction that HFMD would still spread in the city. Countermeasures, including surveillance, case diagnosis, case isolation, vaccination of EV71 vaccine, hand hygiene, social distance, and health promotion, should be strengthened to control and prevent the disease.

The interaction of the pathogens was complex in Changsha City. The reason of the interaction complexity remained unclear. It might result from the climate (temperature, rainfall, and relative humidity), culture and socioeconomic factors,^[24,27–29]^ which might affect the activity of the pathogens and the mode of contact among individuals.

There was another phenomenon that the results of the interactions among the pathogens are similar between *R*_*asc*_ and *R*_*des*_. After interacting, the transmissibility (*R*_*asc*_ or *R*_*des*_) of the affected pathogen was adjusted and verged to 1. The reason of the phenomenon also remains unknown. Therefore, it is essential to explore the mechanism and the risk factors of the interaction clearly, and more research should be focused on these issues to control and prevent HFMD.

## 5 Limitations

There are several limitations in our study. The first one is that only a part of HFMD cases were confirmed cases so we could not test all the reported clinically diagnosed cases. Although 214,178 HFMD cases (including clinically diagnosed cases and confirmed cases) were reported in Changsha City during the study period, only limited stool or anal swab specimens were collected from the reported cases. However, the Dataset B provided us the trends of the proportion of the 3 pathogens of HFMD during the study period. Combined with the data of the reported cases, the number of EV71, CA16, and Other cases could be adjusted. Therefore, the combined data provided us a possibility of analyzing the interaction of the transmissibility of main pathogens of HFMD.

The second limitation is that we could not test and classify the other enteroviruses due to the unavailable data. Although this study aimed to estimate the interaction among the main pathogens of HFMD, more researches are needed to perform the classification of the other pathogens.

Another limitation is that we did not consider cross immunity and coinfection among different pathogens, which might affect our results.

## 6 Conclusions

The reported number of HFMD increased but the reported fatality decreased yearly in Chang City, China. The model we developed could be used for estimating the transmissibility of HFMD. The interaction of the pathogens existed in the city. The effective reproduction number of the affected pathogen was adjusted and verges to 1 by the interaction.

## Acknowledgments

The authors thank all study participants for providing the data and field investigators for collecting the data.

## Author contributions

**Conceptualization:** Shixiong Hu, Benhua Zhao, Lidong Gao, Tianmu Chen.

**Data curation:** Kaiwei Luo, Jia Rui, Shixiong Hu, Dong Yang, Shan Xiao, Yao Wang, Xingchun Liu, Lili Pan, Ran An, Dongbei Guo, Yanhua Su, Lidong Gao.

**Formal analysis:** Kaiwei Luo, Jia Rui, Shixiong Hu, Qingqing Hu, Zeyu Zhao, Yao Wang, Xingchun Liu, Lili Pan, Ran An, Dongbei Guo, Yanhua Su, Tianmu Chen.

**Funding acquisition:** Shixiong Hu, Benhua Zhao, Lidong Gao, Tianmu Chen.

**Investigation:** Kaiwei Luo, Shixiong Hu, Dong Yang, Shan Xiao, Lidong Gao.

**Methodology:** Jia Rui, Zeyu Zhao, Tianmu Chen.

**Project administration:** Shixiong Hu, Benhua Zhao, Tianmu Chen.

**Resources:** Kaiwei Luo, Dong Yang, Shan Xiao, Benhua Zhao, Lidong Gao, Tianmu Chen.

**Software:** Jia Rui, Zeyu Zhao, Tianmu Chen.

**Supervision:** Benhua Zhao, Lidong Gao, Tianmu Chen.

**Validation:** Tianmu Chen.

**Visualization:** Tianmu Chen.

**Writing – original draft:** Qingqing Hu, Tianmu Chen.

**Writing – review & editing:** Qingqing Hu, Tianmu Chen.

Tianmu Chen orcid: 0000-0003-0710-5086.
